# Stress, Coping, and Psychiatric Symptoms in Pregnant Women in Outpatient Care During the 2021 Second-Wave COVID-19 Pandemic

**DOI:** 10.3389/fpsyt.2021.775585

**Published:** 2022-01-06

**Authors:** Chiara Penengo, Chiara Colli, Maddalena Cesco, Veronica Croccia, Matilde Degano, Alessandra Ferreghini, Marco Garzitto, Marci Lobel, Heidi Preis, Alessia Sala, Lorenza Driul, Matteo Balestrieri

**Affiliations:** ^1^Unit of Psychiatry, Department of Medicine (DAME), University of Udine, Udine, Italy; ^2^Obstetric-Gynecologic Clinic, Department of Medicine (DAME), University of Udine, Udine, Italy; ^3^Department of Psychology, Stony Brook University, Stony Brook, NY, United States

**Keywords:** COVID-19, pregnancy, prenatal stress, coping, anxiety, depression

## Abstract

**Aims:** Women face many sources of stress throughout their lives, and some periods are particularly sensitive; pregnancy is one of them. The COVID-19 pandemic is a likely source of additional stress for pregnant women. Moreover, there is evidence that pregnant women have experienced high levels of anxiety and depression symptoms during the pandemic. Our study aimed to evaluate the association of pregnancy-specific stress, pandemic-related stress, and coping strategies with anxiety, depressive and obsessive-compulsive symptomatology in Italian women during the second wave of the COVID-19 pandemic (December 2020–June 2021). We also investigated whether there were differences in these levels of psychopathology compared to a prior study conducted during the first pandemic wave (April–August 2020) in Italian pregnant women.

**Methods:** We assessed 325 pregnant women receiving outpatient prenatal care, using the Revised Prenatal Distress Questionnaire (NuPDQ), Pandemic-Related Pregnancy Stress Scale (PREPS), the Revised Prenatal Coping Inventory (NuPCI), General Anxiety Disorder-7 (GAD-7), Patient Health Questionnaire-2 (PHQ-2), and Obsessive-Compulsive Disorder (OCD) screening. The main analysis was conducted comparing multiple logistic regression models predicting each psychopathological outcome from specific covariates and NuPDQ, PREPS, and NuPCI scores.

**Results:** 42.8% of the sample reported significant levels of anxiety, while 10.3% was positive on depression screening and 13.1% on OCD screening. No significant difference was found in the prevalence of high anxiety, depression, or OCD screening scores compared with the first pandemic wave. Controlling for covariates, we found that GAD-7 and PHQ-2 scores were predicted by pregnancy-specific stress; positive OCD screening was not. The model of high anxiety was improved by adding pandemic-related stress as a predictor (in particular, feeling unprepared for delivery and postpartum). Finally, coping strategies (avoidance, spiritual coping, and planning-preparation) significantly improved prediction of all three psychopathological outcomes.

**Conclusions:** The present study suggests the importance of pregnancy-related stress, COVID-19 pandemic stress, and of coping strategies in counteracting or contributing to psychiatric symptomatology during the current pandemic.

## Introduction

Coronavirus disease (COVID-19) is considered one of the greatest public health crises since the SARS outbreak in 2003 and was declared a global pandemic by the World Health Organization (WHO) on March 11th 2020 (1).

It is now well-known that a pandemic is not only dangerous for physical health but also has great impact on psychological well-being. Various studies over the last year focused on the consequences of the COVID-19 pandemic for stress, anxiety, and depression in the general population (2–4) and in pregnant women (5–9). For example, in a study conducted in the general population, between 17 and 36% of the sample exhibited stress levels that ranged from moderate to severe (10). A recent systematic review (11) concluded that the prevalence of overall stress was highly variable, between 8.1 and 81.9% in general population studies.

Similarly, high levels of prenatal distress, especially anxiety and depression, have been reported in recent systematic reviews and meta-analyses (12–14). The prevalence of depressive symptoms in pregnant women during the COVID-19 pandemic has been reported to be between 25 and 30%, and the prevalence of anxiety has been estimated between 34 and 42% (13, 14), although significant heterogeneity exists across studies. The prevalence of psychological distress was found to vary in the range of 63–70% (15). Other studies indicate that these symptoms were more frequent during the pandemic than before (16, 17).

Moreover, various studies observed that women develop more severe psychopathological symptoms related to the stressful conditions of the ongoing pandemic than men (10, 18, 19), and a recent longitudinal study found that pregnant women during quarantine had a more pronounced increase in anxiety and depressive symptoms compared to non-pregnant women (20). Indeed, pregnancy is a period characterized by vulnerability with specific worries about physical symptoms, bodily changes, and concerns (21), that may contribute to the pregnancy-specific stress (PSS). This specific stress related to the pregnancy can be exacerbated by the pandemic-related stress (PRS), that may arise from fear of contagion, social distancing, isolation, and reduced access to healthcare services (22–24). Hence, especially during the COVID-19 pandemic, it is important to assess and monitor the psychological well-being of expectant mothers, because prenatal stress, anxiety, and depression have adverse impact on perinatal, maternal, and infant outcomes (21, 25, 26).

It is also important to focus on strategies that may protect against the development of psychiatric symptomatology, specifically by reducing stress levels. Previous studies (27–29) indicated that coping has favorable influence on distress and psychological well-being in pregnant women. However, few studies examined the influence of coping on psychopathological outcomes during the COVID-19 pandemic. Fullana et al. (30) found that simple proactive coping strategies (e.g., a healthy diet, having some hobbies, having the opportunity to stay outdoors and follow a routine) were associated with lower anxiety and depression in a general population convenience sample. In addition, Rettie and Daniels found that maladaptive coping responses mediated an association between intolerance of uncertainty linked to the COVID-19 pandemic and psychological distress, both in the general population and in vulnerable groups, including pregnant women (31). An additional study evaluated the effects of coping on distress and mental health outcomes in 304 pregnant women (32); the authors found that dysfunctional coping and emotion-focused coping mediated the association of COVID-19 distress with psychopathological outcomes. However, to assess coping, this study did not use an instrument designed for pregnant women, such as the Revised Prenatal Coping Inventory – NuPCI (33), that has been shown to be more appropriate and offer greater validity in evaluating coping in expectant mothers.

The main aim of this study was therefore to evaluate the relative association of stress (related to the pregnancy but also stress related to the pandemic) and coping with clinical outcomes, namely anxiety, depressive and obsessive-compulsive symptoms. The study was conducted with pregnant women in Italy during the second wave of the COVID-19 pandemic (December 2020–June 2021). During this period there was a complete lockdown from 7th to 30th of April throughout Italy, with some small differences between Italian regions with high (red) or moderate (orange) risk of infection. During this period, schooling was maintained in attendance for classes up to sixth grade. In the red zones from seventh grade to high school there was only distance learning; in the orange zones seventh and eighth grade were in attendance. The restaurants were closed at dinner, and it was not possible to cross the borders of Italian regions with high risk of infection (red zones). During the period of the study prenatal outpatient cares were always guaranteed, but, in the face of high levels of contagions, some appointments had to be rescheduled. Moreover, during impatient cares and during labor, women could not be accompanied by their partner.

In a previous study, we reported on prenatal stress and psychopathological outcomes during the first wave (April–August 2020) of the COVID-19 pandemic in Italy (8). Thus, a second aim of the present study was to assess whether there were differences in the magnitude of stress and psychiatric symptoms between the first (8) and second wave of the COVID-19 pandemic in Italian pregnant women.

## Methods

### Participants

This observational cross-sectional study was conducted between December 15th 2020 and June 15th 2021. Three hundred and twenty-five pregnant women receiving outpatient prenatal care at the Gynecology Clinic of the University Hospital of Udine (Italy) were enrolled. Inclusion criteria were age over 18 years old, Italian fluency and current pregnancy. All patients provided written informed consent.

Nine pregnant women were excluded due to missing data. The final sample included 316 patients.

All procedures in this study were performed in accordance with the ethical standards of the 1964 Helsinki Declaration and its subsequent amendments. Approval was granted by the Medical Ethics Committee of Friuli Venezia Giulia region (CEUR-2018-Sper-027-ASUIUD).

### Measures

Socio-demographic and background information, COVID-19 exposure, pregnancy, prenatal care, general medical condition, and psychological well-being status were collected *via* a self-report questionnaire.

The following instruments were also administered.

#### Revised Prenatal Distress Questionnaire

The NuPDQ is the revised version of the Prenatal Distress Questionnaire (34), developed by Lobel and colleagues (35). It includes 17 items assessing Pregnancy Specific Stress (PSS) rated 0 (“Not At All”), 1 (“Somewhat”), or 2 (“Very Often”). Items are summed to produce a PSS score ranging from 0 to 34.

The NuPDQ has good reliability (α = 0.55–0.79) and validity (36). We used the Italian version of the NuPDQ adapted by our research group (37).

#### Pandemic-Related Pregnancy Stress Scale

The Pandemic-Related Pregnancy Stress Scale (PREPS) was developed by Preis et al. (38) to measure stress in pregnant women during the COVID-19 pandemic. It includes 15 items rated from 1 (“Very Little”) to 5 (“Very Much”). The PREPS consists of three scales: Preparedness Stress (PREPS-PS) composed of seven items, Prenatal Infection Stress (PREPS-PIS) composed of five items, and Positive Appraisal (PREPS-PA) composed of three items (38). The PREPS-PS scale assesses feeling unprepared for delivery and postpartum, PREPS-PIS assesses fear of perinatal infection, and PREPS-PA represents a strategy for coping with pandemic-related stress. In this study PREPS-PA was not used, since we administered the Revised Prenatal Coping Inventory.

We used the Italian version of the PREPS that was previously validated by our research group (39). The instrument showed acceptable-to-good internal consistency for the PREPS-PS (Cronbach's α = 0.760), PREPS-PIS (α = 0.857), and PREPS-PA (α = 0.747) scales.

#### Revised Prenatal Coping Inventory

The NuPCI is the revised version of the Prenatal Coping Inventory (34), developed in 2008 (33). This instrument evaluates coping strategies of pregnant women and is composed of 42 items, rated 0 (“Never”), 1 (“Almost Never”), 2 (“Sometimes”), 3 (“Fairly Often”), or 4 (“Very Often”). The NuPCI includes three subscales: Planning-Preparation (NuPCI-PP; 15 items), Avoidance (NuPCI-A; 11 items), and Spiritual-Positive Coping (NuPCI-SPC; 6 items). A high scale score indicates more frequent use of a specific coping strategy (33).

The NuPCI possesses good validity and reliability, including in the Italian population (33, 37).

#### Outcome Measures: Anxiety, Depression, and Obsessive-Compulsive Symptoms

The Generalized Anxiety Disorder (GAD-7) questionnaire was developed by Spitzer and colleagues (40). This instrument assesses frequency of anxiety symptoms over the past 2 weeks and consists of seven items, rated 0 (“Not At All”), 1 (“Several Days”), 2 (“Over Half The Days”), or 3 (“Nearly Every Day”). A score of seven or above is considered of clinical interest (41). In this study, patients were classified according to the cut-off as having either high anxiety (HA) or low anxiety (LA). The GAD-7 has good internal consistency (α = 0.89) (41) and has been validated in pregnant women (42).

The Patient Health Questionnaire (PHQ-2) is a short version of the PHQ-9, developed by Kroenke et al. (43). The questionnaire screens frequency of core symptoms of major depressive disorder over the past 2 weeks and is composed of two items, rated 0 (“Not At All”), 1 (“Several Days”), 2 (“Over Half The Days”), or 3 (“Nearly Every Day”). A total score of three or above is considered the cut-off for clinical depression (43). In this study, patients were classified according to the cut-off as having high depression (HD) or low depression (LD). The PHQ-2 has good internal consistency (α = 0.83) (44, 45).

The Screening for obsessive-compulsive symptoms (OCD) is composed of two items from the section “Obsessive-Compulsive Disorder” of the Structured Clinical Interview for DSM-5 (46). Each item is rated “Yes” or “No”; if “Yes” is answered to both questions, the OCD screening was considered positive.

### Data Analysis

To have a uniform metric and to facilitate comparison with previous data, the NuPDQ, NuPCI, and PREPS scales were standardized (converted to z-scores) according to findings reported in previous Italian adaptations (37, 39). The GAD-7 and PHQ-2 scores were also standardized in the current sample.

Continuous measures were summarized using Means (M) and Standard Deviations (SD). Between-group differences were analyzed using a between-group *t*-test with Welch's correction, or the Mann-Whitney's test when the assumption of homogeneity of variance was violated (i.e., resulting from Levene's median-centered test). Similarly, to compare current scores with Italian norms, we used a one-sample *t*-test and Mann-Whitney's test on standardized measures (i.e., comparing with a null mean). The Pearson's product-moment correlation coefficient (r) was also calculated, and its 95% confidence interval (ci). For categorical measures, between-group comparisons were performed using Fisher's exact test or a χ^2^-test. Two-tailed tests were used.

We identified 14 possible covariates: four general variables (age and education in years, low-financial status, recent loss of income); four pregnancy-specific variables (month of pregnancy, high-risk pregnancy, first pregnancy, and planned pregnancy); two well-being measures (life-time emotional/psychiatric problems or psychological/physical abuse and life-time stressful events); and four variables associated with the pandemic (pandemic-related closures in Italy during assessment, COVID-19 diagnosis, rescheduled prenatal care appointments, and living alone).

We fitted a series of multiple logistic regression models to evaluate the prediction of dichotomous symptomatologic outcomes based on cut-offs for anxiety (GAD-7 ≥ 7) and depression (PHQ-2 ≥ 3), and for positive screening to OCD. First, models with covariates only were fitted. Then, predictors of interest were progressively added to each model: pregnancy-specific stress (NuPDQ), pandemic-related stress (PREPS-PS and PREPS-PIS), and coping strategies (NuPCI-PP, NuPCI-A, and NuPCI-SPC). The different models were compared using maximum likelihood-ratio tests, to estimate the statistical significance of each group of predictors and compare their relative contribution to the model. Statistical significance of differences between models, the Bayesian Information Criterion (BIC), and the pseudo coefficient of determination (McFadden's pseudo-R^2^) were calculated and compared. For all models, variance inflation factors were also calculated, but no predictor showed a tolerance below 0.5.

In calculating scale scores, we used mean-substitution for more than two missing items on the NuPDQ, NuPCI-PP, and NuPCI-A scales and for more than one item on the GAD-7, PREPS-PS, PREPS-PIS, and NuPCI-SPC scales. No omissions were allowed for the PHQ-2. In data presentation and preliminary analyses, we used pair-wise deletion of missing data. In order to maximize the number of observations, the reported regression analyses were conducted imputing missing values for covariates. The random forest algorithm was used for imputation (with 100 maximum number of iterations and 1,000 trees), preferring a non-parametric method suited for mixed-type data (47). Analyses were also replicated using list-wise deletion of missing data, without producing meaningful differences in results.

The level of statistical significance was set at α = 0.05. For the preliminary between-group comparison analyses, the results were presented without corrections and then checked by considering 66 (i.e., 23 measures for three symptoms, excluding repetitions) independent multiple comparisons. The Benjamini and Hochberg's method based on False Discovery Rate (FDR) was preferred, considering surviving the correction the *p*-values below 0.00076. For correlation analyses, we controlled results considering 52 comparisons with FDR, accepting a *p* < 0.00096. All results that were not statistically significant after FDR correction were marked in the text (with n.s. after FDR correction) and tables.

All analyses were conducted using R, version 4.1.1 (48).

## Results

### Sample Description

A majority (69.6%) of the 316 participants completed study assessments during the national lockdown/closure period related to the COVID-19 pandemic emergency. Most were in their third trimester of pregnancy (70.6%) and 44.3% of them were pregnant for the first time (56.5% were without children). Almost three-quarters (72.5%) indicated that their pregnancy was planned. Almost a third (28.43%) of participants had a high-risk pregnancy; 3.59% were uncertain of their pregnancy risk, and thus excluded from the multivariate analyses. Participants were between 19 and 46 years old and indicated a medium to high level of education (none of the participants had less than middle-school qualification). Only one reported no current relationship; 6.1% of participants reported living alone. Most of the sample (66.8%) lived in rural areas (i.e., in towns with fewer than 50,000 inhabitants). Prior to the COVID-19 pandemic, 83.3% of participants were employed; 49.0% were employed at the time of the current assessment. Medium financial status was reported by 85.9% of the sample, low financial status by 10.4%. Approximately a third (32.6%) indicated a recent loss of income. A health status between “Good” and “Excellent” was indicated by 83.1% of participants, 19.3% indicated that they have a chronic medical condition. A COVID-19 diagnosis was reported by 11.1% of participants (86.6% of these were during pregnancy), including 5.7% that involved hospitalization. Of the undiagnosed women, 10.4% reported that they may have had COVID-19 but were uncertain. Most participants indicated that they follow pandemic recommendations “Very carefully” (85.6%) and 68.6% were “Interested” or “Very interested” in vaccination after pregnancy. Almost half (48.3%) of participants had their most recent prenatal care appointment in the last week, 33.7% in the last month; two women had an appointment more than 3 months prior to their study participation. Prenatal care appointments were rescheduled due to the public health emergency in 10.7% of cases.

Description of the clinical scales and of the covariates of interest is provided in Table 1.

**Table 1:** Sample sociodemographic and clinical description (*N* = 316).

				**High vs. low scores:**		
**Variable**	***N* (missing %)**	**M ± SD**	**(min, max)**	**GAD-7**	**PHQ-2**	**OCD**
**GAD-7** score	313 (0.9%)	6.52 ± 4.687	(0.00, 21.00)	–	H > L^***^	H > L^***^
High anxiety: score ≥ 7	134	–	–	–	H > L^***^	H > L^***^
z-score	313 (0.9%)	−0.01 ± 0.998	(−1.40, 3.08)	–	–	–
**PHQ-2** score	311 (1.6%)	0.97 ± 1.319	(0.00, 6.00)	H > L^***^	–	H > L^**^^§^
High depression: score≥3	32	–	–	H > L^***^	–	H > L^**^
z-score	311 (1.6%)	0.01 ± 1.007	(-0.73, 3.85)	–	–	–
**OCD**	306 (3.2%)	–	–	–	–	–
Positive screening	40	–	–	H > L^***^	H > L^***^	–
**NuPDQ** score	300 (5.1%)	11.28 ± 5.565	(0.00, 27.00)	H > L^***^	H > L^***^^§^	ns
z-score	297 (6.0%)	−0.15 ± 1.121	(−2.58, 3.00)	–	–	–
**PREPS-PS** score	314 (0.6%)	2.75 ± 0.868	(1.00, 4.71)	H > L^***^	H > L^***^^§^	ns
z-score	314 (0.6%)	−0.14 ± 1.035	(−2.22, 2.20)	–	–	–
**PREPS-PIS** score	316 (0.0%)	2.59 ± 0.998	(1.00, 5.00)	H > L^***^	H > L^**^^§^	ns
z-score	316 (0.0%)	0.05 ± 0.972	(−1.50, 2.40)	–	–	–
**NuPCI-PP** score	306 (3.2%)	2.31 ± 0.604	(0.15, 3.93)	ns	L > H^***^^§^	ns
z-score	303 (4.1%)	0.34 ± 1.002	(−4.55, 2.74)	–	–	–
**NuPCI-A** score	306 (3.2%)	1.12 ± 0.623	(0.00, 3.18)	H > L^***^	H > L^***^	H > L^***^
z-score	303 (4.1%)	0.27 ± 1.052	(−2.35, 3.79)	–	–	–
**NuPCI-SPC** score	305 (3.5%)	1.79 ± 0.949	(0.00, 4.00)	H > L^**^	ns	ns
z-score	302 (4.4%)	0.09 ± 0.982	(−2.00, 2.39)	–	–	–
**Age** (years)	315 (0.3%)	33.25 ± 5.240	(19.00, 46.00)	ns	ns	L > H^*^^§^
**Education** (years)	309 (2.2%)	14.97 ± 3.115	(8.00, 21.00)	ns	ns	ns
**Financial status**	298 (5.7%)	–	–	–	–	–
Low	99	–	–	ns	ns	ns
**Changes in income**	304 (3.8%)	–	–	–	–	–
Recent loss	99	–	–	ns	H > L^*^^§^	ns
**Month of pregnancy**	313 (0.9%)	7.41 ± 1.817	(1.00, 9.00)	ns	ns	ns
**Pregnancy risk**	295 (6.6%)	–	–	–	–	–
High-risk	87	–	–	ns	ns	ns
**Previous pregnancies**	314 (0.6%)	–	–	–	–	–
1st pregnancy	139	–	–	ns	ns	ns
**Planned pregnancy**	306 (3.2%)	–	–	–	–	–
Planned pregnancy	222	–	–	ns	L > H^***^^§^	ns
**Well-being**	316 (0.0%)	–	–	–	–	–
Emotional problems	31	–	–	H > L^*^^§^	H > L^**^	ns
**Life-events**	312 (1.3%)	–	–	–	–	–
Stressors	68	–	–	H > L^*^^§^	ns	ns
**COVID-19 period**	316 (0.0%)	–	–	–	–	–
Lockdown	96	–	–	ns	ns	ns
**COVID-19 diagnosis**	315 (0.3%)	–	–	–	–	–
Positive	35	–	–	ns	ns	ns
**Care appointment**	291(7.9%)	–	–	–	–	–
Rescheduled	31	–	–	H > L^*^^§^	ns	ns
**Housing**	310 (1.9%)	–	–	–	–	–
Lives alone	19	–	–	ns	ns	ns

*Statistically significant differences in GAD-7 scores (≥7) and PHQ-2 scores (≥3) and positive screening for OCD are displayed*.

*GAD-7, General Anxiety Disorder – 7 questionnaire; H, Scores above the cut-off for GAD-7/PHQ-2 or positive screening for OCD; L, Scores below the cut- off for GAD-7/PHQ-2 or negative screening for OCD; M, Mean; Max, Maximum observed value; min, Minimum observed value; N, Number of observations; NuPCI, Revised Prenatal Coping Inventory; NuPCI-A, NuPCI, Avoidance scale; NuPCI-PP, NuPCI, Planning-Preparation scale; NuPCI-SPC, NuPCI, Spiritual-Positive coping scale; NuPDQ, Revised Prenatal Distress Questionnaire; OCD, Obsessive-Compulsive problems (screening); PHQ-2, Patient Health Questionnaire – 2; PREPS, Pandemic-Related Pregnancy Stress questionnaire; PREPS-PIS, PREPS, Perinatal Infection Stress scale; PREPS-PS, PREPS, Preparedness Stress scale; SD, Standard deviation; ns, Not significant*.

* *p <0.050*.

** *p <0.010*.

*** *p <0.001*.

§ *Not statistically significant after FDR correction*.

### Psychopathological Symptoms

Life-time emotional/psychiatric problems were reported by 8.1% of the sample (15 indicated anxiety problems, 9 indicated depressive problems, 7 “other” problems), emotional or physical abuse was reported by 2.7%, and stressful life events were reported by 21.8%.

A total of 42.8% of participants had a HA. A HD was reported by 10.3% of participants (i.e., PHQ-2 ≥ 3). With respect to OCD symptoms, 13.1% of screenings were positive (i.e., indicated both obsessions and compulsions). GAD-7 and PHQ-2 scores were highly and positively correlated [r = +0.686, ci: (+0.62, +0.74), *p* < 0.001]. Participants with HA had scores associated with HD [21.6 vs. 1.69%; odds ratio (95% ci): 15.896 (4.75, 83.49); *p* < 0.001], and more of them were positive for OCD screening [21.4 vs. 6.90%; 3.654 (1.71, 8.26); *p* < 0.001]. On the other hand, participants with HD showed just a trend of association with positivity to OCD screening [35.5 vs. 10.66%; 0.219 (0.09, 0.56); *p* = 0.001, n.s. after FDR correction]. Participants with a positive screening for OCD also had higher scores on the GAD-7 [9.78 ± 4.933 vs. 6.04 ± 4.425; *t*_(48.9)_ = +4.52, *p* < 0.001] and a similar trend was observed on the PHQ-2 (1.65 ± 1.762 vs. 0.86 ± 1.210; U = 3,886.5, *p* = 0.004, n.s. after FDR correction).

Comparing the prevalence of psychiatric symptoms to previous findings from a similar sample, as reported by our research team approximately a year earlier during the first wave of the pandemic (39), there was no significant difference in the magnitude of GAD-7 scores [*t*_(500.6)_ = +1.43, *p* = 0.154] or PHQ-2 scores [*t*_(515.6)_ = +0.36, *p* = 0.716]. Also, the proportion screening positive for OCD was not significantly different for the two samples [Wave 1 sample: 12.4%; 1.063 (0.62, 1.86), *p* = 0.896].

### Stress and Coping Measures

Differences in scores and in selected covariates associated with higher GAD-7 and PHQ-2 scores and with positive screening for OCD are reported in Table 1. Correlations are reported in Supplementary Table 1.

Pregnancy-specific stress as measured by the NuPDQ was positively correlated with GAD-7 scores [r (95% ci): +0.483 (+0.39, +0.57), *p* < 0.001] and with PHQ-2 scores [+0.343 (+0.24, +0.44), *p* < 0.001]. Symptoms scales were also associated with pandemic-related stress: PREPS-PS [GAD-7: +0.408 (+0.31, +0.50), *p* < 0.001; PHQ-2: +0.254 (+0.14, +0.36), *p* < 0.001] and partially with PREPS-PIS [GAD-7: +0.329 (+0.22, +0.43), *p* < 0.001; PHQ-2: +0.156 (+0.04, +0.27), *p* = 0.008, n.s. after FDR correction]. NuPDQ scores were positively correlated with PREPS-PS [+0.490 (+0.40, +0.57), *p* < 0.001] and PREPS-PIS [+0.407 (+0.31, +0.50), *p* < 0.001].

In terms of the coping measures, GAD-7 scores were positively correlated with A [+0.628 (+0.55, +0.69), *p* < 0.001] and with NuPCI-SPC [+0.138 (+0.02, +0.25), *p* = 0.020] scale scores. In addition, the PHQ-2 score was positively associated with NuPCI-A [+0.535 (+0.45, +0.61), *p* < 0.001] and a negative trend was present with NuPCI-PP [−0.159 (−0.27, −0.04), *p* = 0.007, n.s. after FDR correction] scale scores. All three NuPCI scales and the two PREPS scales were correlated (ranging from +0.194 and +0.305), although the correlation between PREPS-PIS and NuPCI-SPC [+0.194, (+0.080, +0.303), *p* = 0.001] was not statistically significant after FDR correction. Finally, NuPDQ scores were positively correlated with A [+0.487 (+0.39, +0.57), *p* < 0.001] and -before FDR correction- with NuPCI-PP [+0.140 (+0.02, +0.25), *p* = 0.018] scale scores.

Comparing current pandemic stress with scores reported from a similar sample during Wave 1 of the pandemic (39), PREPS-PS scale scores were lower in the current study [M ± SD in z-scores: −0.14 ± 1.035; *t*_(313)_ = −2.38, *p* = 0.018], PREPS-PIS scale scores did not differ [+0.05 ± 0.972; *t*_(315)_ = +0.98, *p* = 0.326]. No statistically significant differences between Wave 1 and 2 were observed for the NuPDQ score either [0.06 ± 1.097, *t*_(299)_ = −0.88, *p* = 0.379].

### Regression Analyses

Table 2 displays comparisons between different multiple logistic regression models. Details about all the predictors of the models with covariates only and in final/complete models are reported in Supplementary Tables 2, 3.

**Table 2:** Multiple logistic regression models with maximum likelihood-ratio comparisons.

**High anxiety (GAD-7 ≥ 7)**	**BIC**	**Pseudo-R^**2**^**	**Δ%**	**χ^2^-test**
Null model	+395.340	–	–	–
Covariates only	+443.559	0.079	+7.95%	*p* = 0.006^*^
Covariates + PSS	+387.603	0.238	+15.81%	*p* < 0.001^*^
Covariates + PSS + PRS	+388.733	0.264	+2.61%	*p* = 0.006^*^
Covariates + PSS + PRS + Cope	+367.497^§^	0.362	+9.80%	*p* < 0.001^*^
**High depression (PHQ-2** **≥** **3)**
Null model	+193.355	–	–	–
Covariates only	+229.013	0.232	+23.19%	*p* < 0.001^*^
Covariates + PSS	+225.951	0.278	+4.64%	*p* = 0.003^*^
Covariates + PSS + PRS	+232.716	0.303	+2.42%	*p* = 0.103
Covariates + PSS + PRS + Cope	+205.558^§^	0.538	+23.51%	*p* < 0.001^*^
**Positive screening for OCD**
Null model	+233.488	–	–	–
Covariates only	+294.631	0.079	+7.92%	*p* = 0.205
Covariates + PSS	+300.221	0.079	+0.03%	*p* = 0.797
Covariates + PSS + PRS	+307.186	0.099	+1.91%	*p* = 0.114
Covariates + PSS + PRS + Cope	+311.425^§^	0.154	+5.59%	*p* = 0.005^*^

*Best models for BIC were marked in the BIC column*.

*BIC, Bayesian Information Criterion; Cope, Coping strategies (measured with NuPCI-PP, NuPCI-A, and NuPCI-SPC scales); GAD-7, General Anxiety Disorder – 7 questionnaire; NuPCI, Revised Prenatal Coping Inventory; NuPCI-A, NuPCI, Avoidance scale; NuPCI-PP, NuPCI, Planning-Preparation scale; NuPCI-SPC, NuPCI, Spiritual-Positive coping scale; NuPDQ, Revised Prenatal Distress Questionnaire; OCD, Obsessive-Compulsive problems (screening); PHQ-2, Patient Health Questionnaire – 2; PREPS, Pandemic-Related Pregnancy Stress questionnaire; PREPS-PIS, PREPS, Perinatal Infection Stress scale; PREPS-PS, PREPS, Preparedness Stress scale; PRS, Pandemic-Related Stress (measured with PREPS-PS and PREPS-PIS scales); Pseudo-R^2^, McFadden's pseudo coefficient of determination of the model; PSS, Pregnancy-Specific Stress (measured with the NuPDQ); Δ%, Increase in the percentage of variance explained (compared to the previous model)*.

* *p <0.050*.

§ *Best model (considering statistical significance and BIC)*.

Models with covariates of interest were only statistically significant for high anxiety (GAD-7 ≥ 7) [$\chi_{\left(14\right)}^{2}$ = 30.97, *p* < 0.006] and depression (PHQ-2 ≥ 3) [$\chi_{\left(14\right)}^{2}$ = 43.53, *p* < 0.001], but not for positive screening for OCD [$\chi_{\left(14\right)}^{2}$ = 18.04, *p* = 0.205]. Clinical anxiety probability increased with high-risk pregnancy status [odd ratio (95% ci): 1.848 (1.05–3.26), *p* = 0.032] and stressful life-events [1.853 (1.01–3.43), *p* = 0.048]; in contrast, greater age [0.936 (0.88–0.99), *p* = 0.020] and first pregnancy [0.554 (0.32–0.95), *p* = 0.034] were associated with lower probability of high anxiety. Age also had a protective effect for clinically relevant PHQ-2 scores [0.894 (0.81–0.98), *p* = 0.026] together with planned pregnancy [0.284 (0.11–0.70), *p* = 0.006]. In contrast, emotional/psychiatric problems or emotional/physical abuse [5.751 (1.73–19.00), *p* = 0.004] and a previous COVID-19 diagnosis [3.310 (0.97–10.56), *p* = 0.046] were associated with increased risk of high depression. In the complete models, first pregnancy maintained its association with high anxiety [0.475 (0.23–0.94), *p* = 0.036] and high PHQ-2 scores were predicted by diagnosis of COVID-19 [6.714 (1.17–41.49), *p* = 0.033] and by high-risk status [0.129 (0.02–0.53), *p* = 0.009]. In the final model for positive screening for OCD, only age exhibited a protective effect [0.915 (0.84–1.00), *p* = 0.043].

Adding pregnancy-specific stress to the covariate-only models increased model fits for high anxiety and depression, but not for positive screening for OCD.

The model for high anxiety was significantly improved by adding the pandemic-related stress measures (PREPS-PS and PREPS-PIS). However, adding these did not improve the other models.

**Figure 1:**
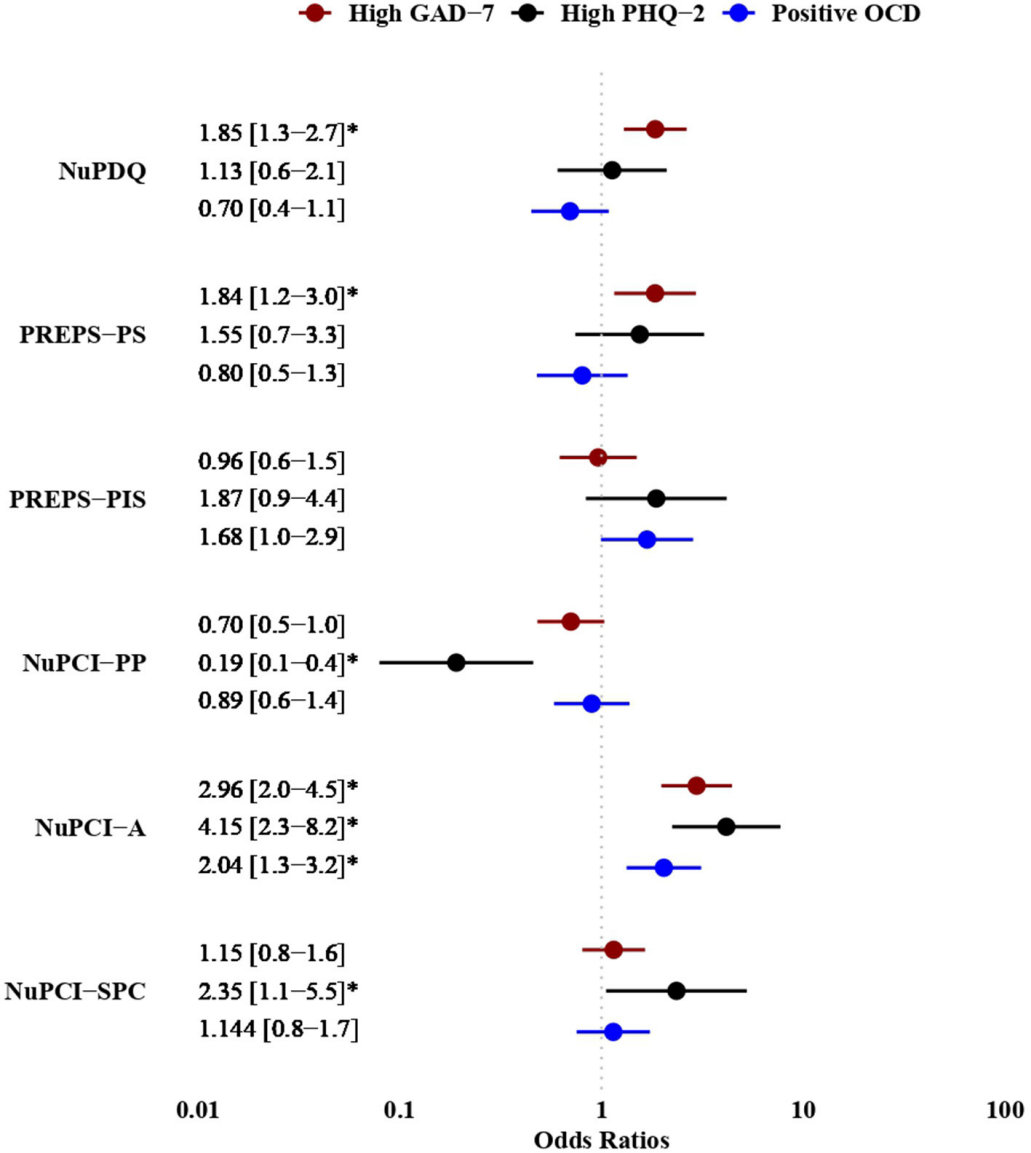
Results of multiple logistic regression models for high anxiety scores (GAD-7 ≥ 7), high depression (PHQ-2 ≥ 3), and positive screening for OCD. Main predictors only are displayed, with corresponding odds ratios and their 95% confidence interval. GAD-7, General Anxiety Disorder – 7 questionnaire; PHQ-2, Patient Health Questionnaire – 2; OCD, Obsessive-Compulsive problems (screening); NuPCI, Revised Prenatal Coping Inventory; NuPCI-A, NuPCI, Avoidance scale; NuPCI-PP, NuPCI, Planning-Preparation scale; NuPCI-SPC, NuPCI, Spiritual-Positive coping scale; NuPDQ, Revised Prenatal Distress Questionnaire; PREPS, Pandemic-Related Pregnancy Stress questionnaire; PREPS-PIS, PREPS, Perinatal Infection Stress scale; PREPS-PS, PREPS, Preparedness Stress scale. *p < 0.050.

Finally, adding coping strategies to the models significantly increased their fit, so that the complete models—including covariates, pregnancy-specific stress, pandemic-related stress, and coping—offered the best prediction of each psychopathological outcome. This was corroborated by BIC scores and Pseudo R^2^ values. Differences in main predictor effects using the final models for high anxiety and depression and for positive screening for OCD are shown in Figure 1. Predicted probabilities for these models are shown in Supplementary Figure 1.

## Discussion

In our study, almost a half of the sample reported high anxiety levels (42.8% of participants), 10.3% of the sample had clinically relevant depression scores, and a significant percentage was positive for screening obsessive-compulsive problems (13.1%). Various studies evaluated the prevalence of psychopathological symptoms during COVID-19 pandemic in pregnant women: recent systematic reviews and meta-analyses observed the prevalence of depressive symptoms between 25 and 30% and the one of anxiety symptoms between 34 and 42% (13, 14). Concerning OCD symptoms, some studies assessed its prevalence in pregnant women during COVID-19 pandemic between 7.12 (49) and 10.3% (50), in line with our study.

A number of findings from this study of Italian women pregnant during the second wave of the pandemic corroborate prior research identifying maternal risk factors for poor mental health, including financial loss (6, 9, 51), unplanned pregnancy (52), and younger age (53). Previous emotional/psychiatric problems and life-time stressful events were also associated with higher levels of psychopathological symptoms, consistent with studies that have established a similar connection both during the COVID-19 pandemic (54, 55) and before (52). Disruption of prenatal care, a distinctive stressor occurring during the pandemic, was also found to contribute to poorer maternal mental health in this study, as it did in a recent cross-national study of pregnant women (56).

The main aim of this study was to evaluate the relative value of pregnancy-specific stress, pandemic-related stress, and coping strategies in predicting each of the psychopathological outcomes, despite their comorbidity. We also examined whether there were differences in the prevalence of psychopathology between the first and second pandemic waves and found that levels of anxiety, depression, and OCD remained stable over this period. Moreover, we found no change in pregnancy-related stress and in pandemic-related stress pertaining to women's fears of perinatal infection, although stress related to feeling unprepared for birth and the post-partum was lower during the second wave of the pandemic. A recent general population study from Germany (57) found sustained high psychological burden over a similar period of time, but documented increased depressive symptoms at the latter timepoint.

In bivariate analyses, both pregnancy-specific stress and stress due to the pandemic (related to feeling unprepared for childbirth and the post-partum and concerns about risk of perinatal infection) were associated with higher rates of anxious and depressive symptoms. Prior research (58, 59) established that prenatal stress is correlated with elevated levels of anxiety and depression, including in Italian women during the first wave of the pandemic (8).

Bivariate analyses also examined the ways of coping that pregnant women adopted to manage pregnancy-specific stress during the pandemic. We found that the ability to plan and prepare was strongly associated with less depressive symptomatology. Such coping strategies can help women to successfully overcome and solve difficult situations (38). Conversely, frequent use of avoidance as a coping strategy was associated with higher levels of all forms of psychopathology. Avoidance is widely recognized as a maladaptive form of coping (30–32, 60). We also found that spiritual-positive coping was associated with greater anxiety. There is some evidence that coping *via* religiosity and spirituality can have both positive and negative impact (61), especially in vulnerable groups such as pregnant women (62).

In evaluating the relative importance of psychopathology predictors in multivariate models, all the outcomes were best explained by models including unique covariates, pregnancy-specific stress, pandemic-related stress, and coping strategies. However, there were differences in the particular covariates predicting each outcome. Concerning the complete models, from all of the covariates examined, only having a prior COVID-19 diagnosis was associated with greater likelihood of depression, whereas older maternal age was the single maternal covariate related to lower likelihood of OCD. Being pregnant for the first time seemed to lower likelihood of high anxiety, an unexpected finding given other research indicating that primigravida have been more likely to experience anxiety during the pandemic (63).

There were similarities in the role of stress variables for depression and anxiety in the present study. Pregnancy-specific stress, for example, was a major contributor to anxiety, whereas pandemic-related stress, although statistically significant, was not a strong contributor to this outcome. Similarly, regarding depressive symptomatology, we found that the inclusion of pandemic-related stress did not significantly improve prediction beyond a model in which pregnancy-specific stress and covariates were the sole included. These findings suggest that pregnancy-specific stress, which originates from worries about physical symptoms, concerns about fetal health, and fears about impending childbirth, may be a particularly important risk factor for maternal anxiety and depression during the current pandemic.

Finally, coping played an especially important role in determining depressive symptomatology in this study, independently explaining almost 24% of variance in depression symptoms. Yet it explained only 9.8% of variance in anxiety, and even less (5.6%) variance in OCD. Of the three outcomes examined in this study, positive screening for obsessive-compulsive symptomatology was the least well-predicted by stress and coping, and only the model including all sets of predictors was statistically significant and achieved acceptability. It seems that in the case of OCD it is the way of stress management, rather than its intensity, that indicates vulnerability. Arguably, in the case of OCD, other aspects of risk come into play that are not accurately captured by measures of pregnancy and pandemic stress.

### Limitations and Strengths

One of the limitations of the study is its cross-sectional design. Hence, we cannot establish causal relationships between stress, coping and symptoms. Another limitation is the use of self-report instruments, which may reduce the validity of responses. However, we used tools with strong psychometric properties, previously validated in Italian, and examined plausible, theoretically informed associations. Additional strengths include the evaluation of different psychopathological dimensions and different types of stress tailored to pregnancy and to the pandemic, as well as assessing the role of coping strategies, which have received little attention in other studies of groups affected by COVID-19.

## Conclusion

The present study reveals substantial stability of psychiatric symptoms experienced by pregnant Italian women across the first two years of the pandemic, despite different conditions and national regulations. The psychiatric symptoms detected in the second year of the pandemic were predicted mainly by perceived pregnancy stress, with relatively less contribution of pandemic related stress. The adoption of coping strategies was also an important factor in counteracting or elevating symptoms.

Study findings underscore the enduring value of alleviating stress in pregnant women to protect their mental health and resultingly, reduce adverse impacts of psychopathology on childbearing women and their offspring. In addition to improving the conditions that create stress during pregnancy, helping pregnant women develop adaptive coping strategies may counteract the development of psychiatric symptoms that can result from high levels of stress, especially during the societal disruption caused by a global pandemic.

## Data Availability Statement

The raw data supporting the conclusions of this article will be made available by the authors, without undue reservation.

## Ethics Statement

The studies involving human participants were reviewed and approved by Medical Ethics Committee of Friuli Venezia Giulia region (CEUR-2018-Sper-027-ASUIUD). The patients/participants provided their written informed consent to participate in this study.

## Author Contributions

MB, LD, HP, and ML contributed to the study design, critically reviewed, and revised the manuscript. CP and CC contributed interpreting data and wrote the manuscript. MC, VC, and AF contributed to writing the manuscript. MG contributed to the statistics analyses and writing the manuscript. AS and MD recruited participants and organized the study data. All authors read and approved the final manuscript.

## Conflict of Interest

The authors declare that the research was conducted in the absence of any commercial or financial relationships that could be construed as a potential conflict of interest.

## Publisher's Note

All claims expressed in this article are solely those of the authors and do not necessarily represent those of their affiliated organizations, or those of the publisher, the editors and the reviewers. Any product that may be evaluated in this article, or claim that may be made by its manufacturer, is not guaranteed or endorsed by the publisher.
