# Supplementary material for: Stress, Coping, and Psychiatric Symptoms in Pregnant Women in Outpatient Care During the 2021 Second-Wave COVID-19 Pandemic
Source: Front Psychiatry. 2022 Jan 6;12:775585. doi: 10.3389/fpsyt.2021.775585 (PMC8775005; doi:10.3389/fpsyt.2021.775585)
Supplement: Supplementary file 1 [file Data_Sheet_1.docx]

**Table S1:** Pearson’s correlations between scales and scales and continuous covariates (with their 95% confidence interval and statistical significances).

|  | **GAD-7** |  |  |  |  |  |  |  |
| --- | --- | --- | --- | --- | --- | --- | --- | --- |
| **PHQ-2** | +0.686  [+0.619, +0.743]  p<0.001*** | **PHQ-2** |  |  |  |  |  |  |
| **NuPDQ** | +0.483  [+0.388, +0.567]  p<0.001*** | +0.343  [+0.236, +0.441]  p<0.001*** | **NuPDQ** |  |  |  |  |  |
| **PREPS-PS** | +0.408  [+0.306, +0.500]  p<0.001*** | +0.254  [+0.142, +0.359]  p<0.001*** | +0.490  [+0.397, +0.573]  p<0.001*** | **PREPS-PS** |  |  |  |  |
| **PREPS-PIS** | +0.329  [+0.222, +0.429]  p<0.001*** | +0.156  [+0.040, +0.267]  p=0.008**§ | +0.407  [+0.305, +0.499]  p<0.001*** | +0.636  [+0.562, +0.701]  p<0.001*** | **PREPS-PIS** |  |  |  |
| **NuPCI-PP** | -0.045  [-0.160, +0.071]  p=0.449 | -0.159  [-0.270, -0.044]  p=0.007**§ | +0.140  [+0.024, +0.252]  p=0.018*§ | +0.196  [+0.081, +0.305]  p=0.001** | +0.205  [+0.091, +0.314]  p<0.001*** | **PREPS-PP** |  |  |
| **NuPCI-A** | +0.628  [+0.553, +0.694]  p<0.001*** | +0.535  [+0.447, +0.613]  p<0.001*** | +0.487  [+0.393, +0.570]  p<0.001*** | +0.305  [+0.196, +0.407]  p<0.001*** | +0.265  [+0.154, +0.370]  p<0.001*** | +0.162  [+0.047, +0.273]  p=0.006**§ | **NuPCI-A** |  |
| **NuPCI-SPC** | +0.138  [+0.022, +0.250]  p=0.020*§ | +0.057  [-0.059, +0.172]  p=0.334 | +0.098  [-0.018, +0.211]  p=0.099 | +0.208  [+0.094, +0.316]  p<0.001*** | +0.194  [+0.080, +0.303]  p=0.001**§ | +0.223  [+0.110, +0.330]  p<0.001*** | +0.221  [+0.108, +0.329]  p<0.001*** | **NuPCI-SPC** |
| **Age**  *(years)* | -0.059  [-0.174, +0.057]  p=0.318 | -0.116  [-0.229, +0.000]  p=0.051 | -0.042  [-0.157, +0.074]  p=0.477 | -0.187  [-0.296, -0.072]  p=0.002**§ | -0.075  [-0.189, +0.042]  p=0.207 | -0.134  [-0.246, -0.018]  p=0.024*§ | -0.110  [-0.223, +0.006]  p=0.062 | -0.082  [-0.196, +0.034]  p=0.166 |
| **Schooling**  *(years)* | +0.041  [-0.075, +0.156]  p=0.487 | -0.056  [-0.171, +0.061]  p=0.348 | -0.017  [-0.133, +0.099]  p=0.774 | -0.016  [-0.132, +0.100]  p=0.784 | +0.037  [-0.079, +0.153]  p=0.530 | +0.044  [-0.073, +0.159]  p=0.462 | +0.015  [-0.101, +0.131]  p=0.795 | -0.045  [-0.160, +0.072]  p=0.452 |
| **Month of**  **pregnancy** | -0.042  [-0.157, +0.074]  p=0.478 | -0.097  [-0.210, +0.020]  p=0.103 | +0.026  [-0.090, +0.142]  p=0.660 | +0.040  [-0.076, +0.156]  p=0.497 | +0.077  [-0.039, +0.191]  p=0.193 | +0.410  [+0.309, +0.502]  p<0.001*** | +0.050  [-0.066, +0.165]  p=0.397 | +0.050  [-0.067, +0.165]  p=0.404 |
|  |  |  |  |  |  |  |  |  |
| **GAD-7:** General Anxiety Disorder – 7 questionnaire; **NuPCI:** Revised Prenatal Coping Inventory; **NuPCI-A:** NuPCI, Avoidance scale; **NuPCI-PP:** NuPCI, Planning-Preparation scale; **NuPCI-SPC:** NuPCI, Spiritual-Positive coping scale; **NuPDQ:** Revised Prenatal Distress Questionnaire; **PHQ-2:** Patient Health Questionnaire – 2; **PREPS:** Pandemic-Related Pregnancy Stress questionnaire; **PREPS-PIS:** PREPS, Perinatal Infection Stress scale; **PREPS-PS:** PREPS, Preparedness Stress scale.. ***:** Statistically significant with p<0.050; ****:** Statistically significant with p<0.010; *****:** Statistically significant with p<0.001; §: Not statistically significant after FDR correction. | | | | | | | | |

**Table S2.** Multiple logistic regression models for high anxiety scores (GAD-7≥7), high depression (PHQ-2≥3), and positive screening for OCD. Odd-ratios with their 95% confidence interval are reported for the predictors. Only covariates are included as predictors.

|  | **High anxiety** | **High depression** | **Positive for OCD** |
| --- | --- | --- | --- |
| **Model** |  |  |  |
| *BIC* | +443.559 | +229.013 | +294.631 |
| *Pseudo-R^2^* | 0.079 | 0.232 | 0.079 |
| *Statistical significance* | χ^2^(14)=30.97, p<0.006* | χ^2^(14)=43.53, p<0.001* | χ^2^(14)=18.04, p=0.205 |
| **Predictors** |  |  |  |
| ***Covariates*** |  |  |  |
| *Age in years* | 0.936 [0.88-0.99], p=0.020* | 0.894 [0.81-0.98], p=0.026* | 0.904 [0.83-0.98], p=0.013* |
| *Schooling in years* | 1.081 [0.98-1.19], p=0.105 | 0.973 [0.83-1.15], p=0.739 | 1.006 [0.88-1.15], p=0.929 |
| *Financial-status (low =1)* | 2.357 [0.96-5.90], p=0.062 | 1.246 [0.31-4.37], p=0.741 | 0.692 [0.14-2.57], p=0.613 |
| *Changes in economic income (recent loss =1)* | 0.603 [0.33-1.08], p=0.092 | 1.316 [0.48-3.49], p=0.586 | 0.461 [0.18-1.09], p=0.091 |
| *Month of pregnancy* | 1.010 [0.88-1.16], p=0.890 | 0.865 [0.69-1.09], p=0.217 | 0.904 [0.74-1.10], p=0.306 |
| *Pregnancy risk (high-risk =1)* | 1.848 [1.05-3.26], p=0.032* | 0.540 [0.16-1.55], p=0.277 | 2.049 [0.93-4.47], p=0.071 |
| *Previous pregnancies (1^st^ pregnancy =1)* | 0.554 [0.32-0.95], p=0.034* | 0.471 [0.17-1.22], p=0.133 | 0.830 [0.39-1.75], p=0.628 |
| *Planification of pregnancy (planned =1)* | 0.701 [0.39-1.25], p=0.226 | 0.284 [0.11-0.70], p=0.006* | 0.961 [0.43-2.25], p=0.923 |
| *Psychological well-being (problems or abuse =1)* | 2.214 [0.94-5.37], p=0.071 | 5.751 [1.73-19.00], p=0.004* | 2.633 [0.91-7.04], p=0.060 |
| *Life-events (stressor =1)* | 1.853 [1.01-3.43], p=0.048* | 1.610 [0.54-4.48], p=0.371 | 1.068 [0.42-2.54], p=0.885 |
| *COVID-19 period (lockdown =1)* | 1.064 [0.61-1.88], p=0.830 | 1.795 [0.65-5.68], p=0.285 | 0.841 [0.39-1.86], p=0.660 |
| *COVID-19 diagnosis (positive =1)* | 0.746 [0.32-1.68], p=0.485 | 3.310 [0.97-10.56], p=0.046* | 0.587 [0.13-1.92], p=0.426 |
| *Care prenatal appointment (rescheduled =1)* | 2.165 [0.89-5.48], p=0.092 | 3.313 [0.84-11.74], p=0.070 | 2.226 [0.65-6.69], p=0.171 |
| *Housing (lives alone =1)* | 2.679 [0.85-9.09], p=0.098 | 1.642 [0.23-8.63], p=0.582 | 1.680 [0.34-6.45], p=0.478 |
|  |  |  |  |
| **BIC:** Bayesian Information Criterion; **GAD-7:** General Anxiety Disorder – 7 questionnaire; **OCD:** Obsessive-Compulsive problems (screening); **PHQ-2:** Patient Health Questionnaire – 2; **Pseudo-R^2^:** McFadden’s pseudo coefficient of determination of the model. ***:** Statistically significant with p<0.050. | | | |

**Table S3.** Multiple logistic regression models for high anxiety scores (GAD-7≥7), high depression (PHQ-2≥3), and positive screening for OCD. Odd-ratios with their 95% confidence interval are reported for the predictors. Measures of pregnancy-specific stress (NuPDQ scale), of pandemic-related stress (PREPS: PS and PIS scales) and of coping (NuPCI: PP, A, and SPC scales) are included as predictors, considering also covariates.

|  | **High anxiety** | **High depression** | **Positive for OCD** |
| --- | --- | --- | --- |
| **Model** |  |  |  |
| *BIC* | +367.497 | +205.558 | +311.425 |
| *Pseudo-R^2^* | 0.362 | 0.538 | 0.154 |
| *Statistical significance* | χ^2^(20)=140.96, p<0.001* | χ^2^(20)=100.92, p<0.001* | χ^2^(20)=35.182, p=0.019* |
| **Predictors** |  |  |  |
| ***Main predictors*** |  |  |  |
| *NuPDQ* | 1.846 [1.30-2.67], p=0.001* | 1.129 [0.60-2.13], p=0.702 | 0.698 [0.44-1.08], p=0.111 |
| *PREPS-PS* | 1.843 [1.16-2.97], p=0.010* | 1.547 [0.75-3.31], p=0.244 | 0.803 [0.47-1.34], p=0.406 |
| *PREP-PIS* | 0.963 [0.62-1.50], p=0.866 | 1.869 [0.86-4.37], p=0.127 | 1.681 [1.00-2.89], p=0.053 |
| *NuPCI-PP* | 0.705 [0.48-1.02], p=0.072 | 0.191 [0.07-0.42], p<0.001* | 0.895 [0.58-1.38], p=0.612 |
| *NuPCI-A* | 2.963 [2.01-4.52], p<0.001* | 4.154 [2.35-8.23], p<0.001* | 2.037 [1.34-3.17], p=0.001* |
| *NuPCI-SPC* | 1.149 [0.80-1.65], p=0.446 | 2.353 [1.08-5.50], p=0.037* | 1.144 [0.75-1.74], p=0.529 |
| ***Covariates*** |  |  |  |
| *Age in years* | 0.969 [0.90-1.04], p=0.387 | 0.885 [0.76-1.01], p=0.082 | 0.915 [0.84-1.00], p=0.043* |
| *Schooling in years* | 1.061 [0.94-1.20], p=0.331 | 0.805 [0.64-1.00], p=0.057 | 0.976 [0.85-1.13], p=0.734 |
| *Financial-status (low =1)* | 1.492 [0.44-5.12], p=0.519 | 0.338 [0.04-2.20], p=0.288 | 0.606 [0.11-2.52], p=0.524 |
| *Changes in economic income (recent loss =1)* | 0.618 [0.28-1.31], p=0.214 | 2.223 [0.54-9.31], p=0.263 | 0.513 [0.18-1.30], p=0.176 |
| *Month of pregnancy* | 1.071 [0.88-1.31], p=0.492 | 0.964 [0.69-1.36], p=0.826 | 0.896 [0.72-1.12], p=0.333 |
| *Pregnancy risk (high-risk =1)* | 0.899 [0.42-1.87], p=0.777 | 0.129 [0.02-0.53], p=0.009* | 1.703 [0.72-3.99], p=0.219 |
| *Previous pregnancies (1^st^ pregnancy =1)* | 0.475 [0.23-0.94], p=0.036* | 0.961 [0.21-4.13], p=0.958 | 1.007 [0.43-2.37], p=0.988 |
| *Planification of pregnancy (planned =1)* | 1.012 [0.47-2.19], p=0.976 | 0.429 [0.12-1.44], p=0.169 | 1.351 [0.58-3.36], p=0.500 |
| *Psychological well-being (problems or abuse =1)* | 1.713 [0.51-5.76], p=0.379 | 4.539 [0.73-27.98], p=0.098 | 2.184 [0.65-6.81], p=0.188 |
| *Life-events (stressor =1)* | 1.123 [0.49-2.53], p=0.781 | 1.041 [0.21-4.97], p=0.960 | 1.096 [0.39-2.83], p=0.855 |
| *COVID-19 period (lockdown =1)* | 0.899 [0.43-1.88], p=0.777 | 1.384 [0.35-5.91], p=0.647 | 0.753 [0.32-1.81], p=0.518 |
| *COVID-19 diagnosis (positive =1)* | 0.844 [0.28-2.41], p=0.754 | 6.714 [1.17-41.49], p=0.033* | 0.591 [0.12-2.19], p=0.470 |
| *Care prenatal appointment (rescheduled =1)* | 2.133 [0.66-7.10], p=0.208 | 5.212 [0.80-33.23], p=0.075 | 2.000 [0.55-6.39], p=0.260 |
| *Housing (lives alone =1)* | 2.378 [0.50-11.88], p=0.280 | 0.646 [0.01-13.02], p=0.810 | 1.492 [0.28-6.20], p=0.604 |
|  |  |  |  |
| **BIC:** Bayesian Information Criterion; **GAD-7:** General Anxiety Disorder – 7 questionnaire; **NuPCI:** Revised Prenatal Coping Inventory; **NuPCI-A:** NuPCI, Avoidance scale; **NuPCI-PP:** NuPCI, Planning-Preparation scale; **NuPCI-SPC:** NuPCI, Spiritual-Positive coping scale; **NuPDQ:** Revised Prenatal Distress Questionnaire; **OCD:** Obsessive-Compulsive problems (screening); **PHQ-2:** Patient Health Questionnaire – 2; **PREPS:** Pandemic-Related Pregnancy Stress questionnaire; **PREPS-PIS:** PREPS, Perinatal Infection Stress scale; **PREPS-PS:** PREPS, Preparedness Stress scale; **Pseudo-R^2^:** McFadden’s pseudo coefficient of determination of the model. ***:** Statistically significant with p<0.050. | | | |

**Figure S1:** Results of multiple logistic regression models for high anxiety scores (GAD-7≥7), high depression (PHQ-2≥3), and positive screening for OCD. Predicted probabilities for main predictors only are reported.

**
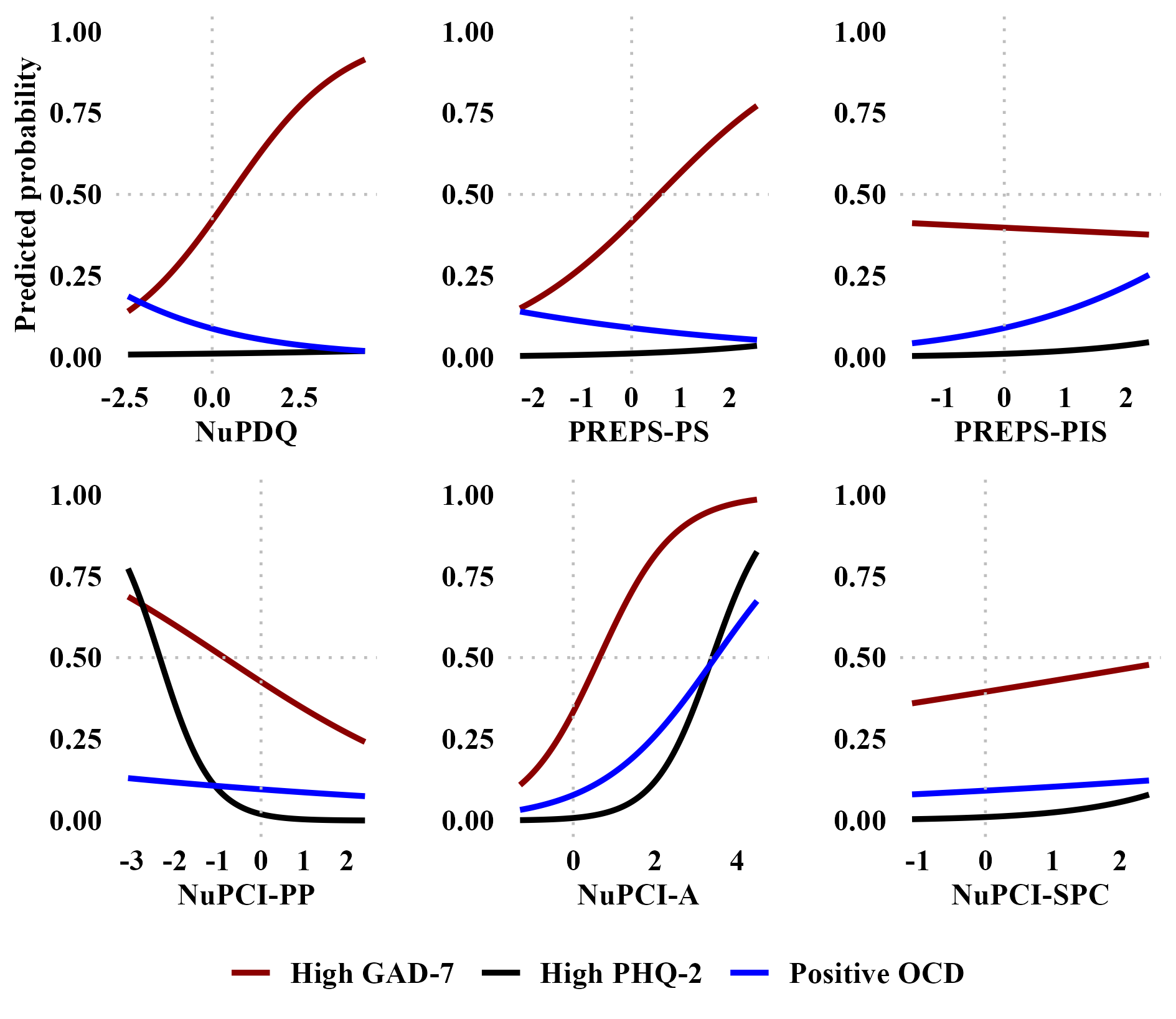
**

**GAD-7:** General Anxiety Disorder – 7 questionnaire; **NuPCI:** Revised Prenatal Coping Inventory; **NuPCI-A:** NuPCI, Avoidance scale; **NuPCI-PP:** NuPCI, Planning-Preparation scale; **NuPCI-SPC:** NuPCI, Spiritual-Positive coping scale; **NuPDQ:** Revised Prenatal Distress Questionnaire; **OCD:** Obsessive-Compulsive problems (screening); **PHQ-2:** Patient Health Questionnaire – 2; **PREPS:** Pandemic-Related Pregnancy Stress questionnaire; **PREPS-PIS:** PREPS, Perinatal Infection Stress scale; **PREPS-PS:** PREPS, Preparedness Stress scale. ***:** Statistically significant with p<0.050.
